# Deeper Exploiting Graph Structure Information by Discrete Ricci Curvature in a Graph Transformer

**DOI:** 10.3390/e25060885

**Published:** 2023-06-01

**Authors:** Xin Lai, Yang Liu, Rui Qian, Yong Lin, Qiwei Ye

**Affiliations:** 1 School of Mathematics, Renmin University of China, Beijing 100872, China; laixin@ruc.edu.cn; 2 Beijing Academy of Artificial Intelligence, Beijing 100084, China; 3 School of Information, Renmin University of China, Beijing 100872, China; 4 Yau Mathematics Science Center, Tsinghua University, Beijing 100084, China

**Keywords:** transformers, discrete Ricci curvature, structure information

## Abstract

Graph-structured data, operating as an abstraction of data containing nodes and interactions between nodes, is pervasive in the real world. There are numerous ways dedicated to extract graph structure information explicitly or implicitly, but whether it has been adequately exploited remains an unanswered question. This work goes deeper by heuristically incorporating a geometric descriptor, the discrete Ricci curvature (DRC), in order to uncover more graph structure information. We present a curvature-based topology-aware graph transformer, termed Curvphormer. This work expands the expressiveness by using a more illuminating geometric descriptor to quantify the connections within graphs in modern models and to extract the desired structure information, such as the inherent community structure in graphs with homogeneous information. We conduct extensive experiments on a variety of scaled datasets, including PCQM4M-LSC, ZINC, and MolHIV, and obtain a remarkable performance gain on various graph-level tasks and fine-tuned tasks.

## 1. Introduction

Graph data include considerable structure information; however, existing graph-based algorithms do not fully use the inherent structural information of graphs. Real-word datasets with an inherent node–edge structure, such as citation networks [1], molecules [2], and the Internet [3], can be naturally represented by graphs. Moreover, graphs can be manually established in scattered data such as point clouds [4,5].

The vast majority of GNNs use a message passing (MP) mechanism to explore the graph structure information by aggregating neighborhood information [6,7,8]; however, they unavoidably run into oversmoothing and oversquashing issues. Due to the MP mechanism, most graph convolution of GNNs may be considered as a special case of Laplacian smoothing [9]. Analogously to random walk on graphs, smoothing operations on graphs result in the mixing of the characteristics of individual nodes.Multiple processes are taken to smooth the characteristics of individual nodes, culminating in the reduction of variability across nodes from diverse groups. This phenomenon of the inability to classify nodes when the network is deeper is the most widely discussed defect of GNNs, i.e., oversmoothing [9,10]. Another newly discussed problem of GNNs is oversquashing [11,12], which indicates that information flows between long-distant nodes encounter an unavoidable distortion. Oversmoothing and oversquashing are inevitable side effects of MP GNNs. Rong et al. [10] alleviated oversmoothing by randomly dropping a percentage of edges in the graph. Alon and Yahav [11] tried to tackle oversquashing by adding a fully adjacent layer. However, these approaches could not totally resolve these issues [13].

Graph-based transformers are another line of recent research. Transformers were originally proposed as powerful solvers for natural language processing (NLP) tasks [14] and soon became prevailing in many domains, such as computer vision [15], time series [16], and graph representation learning [17,18]. For graph-based transformers, current works mainly focus on how to integrate a graph structure into positional encoding (PE) in transformers [18,19]. Since graph data do not have a canonical position as in images and sequences, the most widely used PE is the graph Laplacian eigenvectors, which preserve the global structure with a permutation invariance [20]. Different from PE methods, Graphormer [21] added structural encodings to the self-attention module as a structure-aware bias of attention weights.It has been experimentally proved that Graphormer is exempt from the problem of oversmoothing. Moreover, because of the self-attention mechanism in the transformer architecture, each node in the network attends to the others as if they were entirely nearby nodes. Consequently, transformer-based graph learners can efficiently avoid the issue of oversquashing. Thus, it is natural to take graph transformers as the backbone architecture for graph-based models.

However, current graph structure descriptors, such as node degrees and shortest path distances (SPD), have limited expressiveness. Rich information in the topology of the graphs still remains unexplored. Graph-based tasks rely heavily on structure information. The basic distinction between graph data and other data types, such as pictures or sequences, is the non-Euclidean node–edge structure. Graphs can be treated as a discretized manifold [22] from the topological view. Based on the homophily assumption of most graphs, the mainstream graph-based tasks, such as node classification, link prediction and graph classification/regression, tend in essence to strengthen the connection between nodes with the same property and discriminate against nodes with different properties. To describe the geometric relationships of nodes from intra-/intercommunities, we draw inspiration from recent research focusing on developing community detection algorithms [22,23,24] with the help of a geometric notion, i.e., the discrete Ricci curvature (DRC) [25].

**Figure 1:**
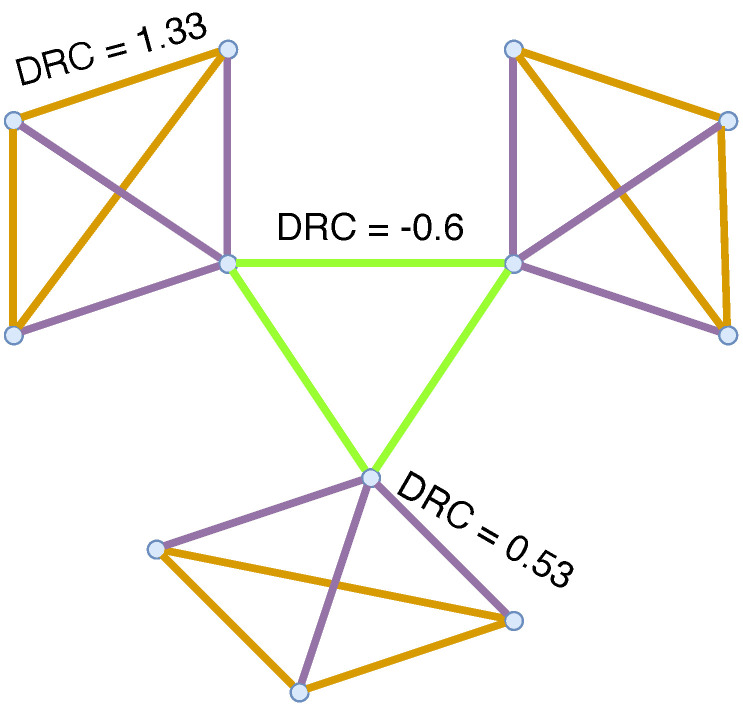
Illustration of DRC on a small graph. Edges with the same color have the same DRC value because of symmetry. Dense connections (yellow edges) correspond to a positive DRC, while sparse connections (green edges) have a negative DRC.

The DRC quantifies the intensity of connections between nodes and their neighborhoods with regard to the local graph topology. Node pairs being densely connected are associated with positive DRC values, while sparsely connected pairs give rise to negative DRC values. As illustrated in Figure 1, the nodes connected by a yellow edge are in the same community and have densely connected/overlapped neighborhoods, while the nodes connected by a green edge are from distinct communities with few connections/overlaps between their neighborhoods. Therefore, the DRC value of the yellow edges is 1.33, which is obviously larger than the value −0.6 of the green edges. Purple edges correspond to a scenario between the two extremes; thus, they have a DRC between −0.6 and 1.33. Intuitively, the DRC has the ability to measure the connectedness of nodes and their neighborhoods, thus it can be integrated to graph transformers to explore deeper structure information.

In this paper, we propose a novel curvature-based topology-aware graph transformer architecture, namely, Curvphormer, to exploit advanced structural information from a topological view. We evaluated the performance of our proposed algorithms on widely used testbeds such as MolHIV, PCQM4M-LSC, and ZINC. Curvphormer exceeded previous benchmarks by a significant margin.

## 2. Related Work

In this section, we highlight the most recent approaches on NN-based models working on demystifying the structure information of graph data. Then, we give prominence to some related applications of the DRC in finding the underlying structure of graphs.

### 2.1. Structural Encodings

#### 2.1.1. On MP-GNNs

GNN methods processing graph data have natural merits from a theoretical basis. Most GNNs follow the MP mechanism and leverage random walk algorithms to explore the underlying structure of graphs with the aid of stochastic theories [9,26]. Some other GNN methods try to incorporate local structure information by utilizing a local *k*-hop subgraph as the structure fingerprint of its central node [27,28]. Moreover, some methods propose to explicitly or implicitly introduce some additional structure information encoded by geometric notions such as DRC to GNNs  [29,30]. However, due to the inevitable oversmoothing and oversquashing problems and the limited expressiveness of GNNs, the increment of structure information does not yield much improvement in performance.

#### 2.1.2. On Graph-Based Transformers

The challenge of building a powerful transformer architecture in graph representation is how to properly encode structure information into a positional encoding (PE) module [18] or the self-attention module [21]. Dwivedi and Bresson[18] exploited graph structure by precomputing the Laplacian eigenvectors of the adjacency matrix acting as the PE in the vanilla transformer architecture to provide distance-aware information. Graph-BERT [19] operates on sampled linkless subgraphs for the local structure information and enhances its capability on extremely large graphs. Furthermore, Graph-BERT introduces three PE embeddings to take in the positional information on local subgraphs. Specifically, a Weisfeiler–Lehman (WL) absolute PE is leveraged to capture the global information, and an intimacy-based PE and a hop-based relative PE are introduced to extract the local information in subgraphs. It is notable that TokenGT [17] puts forward that pure transformers can attain impressive performance on graphs by an orthonormal node identifier and a type identifier. It suggests that the transformer architecture itself has the potential to fit in the graph structure. The key to developing transformers for graphs is to extract proper graph structure information in the model. Thus, most graph transformers incorporate graph structure information by some strong graph-specific modifications. Following this guideline, further involving advanced geometric descriptors into the transformer architecture is a promising direction.

### 2.2. DRC in Finding Graph Structure

In light of the property of the Ricci curvature in Riemannian geometry, the discrete version of the Ricci curvature is a natural choice as a topological descriptor. Ni et al. [3] leveraged the DRC to analyze Internet topologies. Sia et al. [23] constructed a community detection algorithm by removing negative curved edges step by step. Lai et al. [24] leveraged a DRC-based Ricci flow to deform a graph, then intracommunity nodes became closer and intercommunity nodes dispersed. The DRC is capable of finding the underlying relationship between nodes, characterizing them to clusters with identical or distinct properties.

## 3. Method

In this section, we elaborate the formulation of the discrete Ricci curvature (DRC) and how to incorporate it in Curvphormer. Firstly, the basic settings are stated in Section 3.1. Then, we carefully define the Ricci curvature on graphs in Section 3.2. In Section 3.3, we propose the curvature-based topology-aware Curvphormer.

### 3.1. Preliminaries

  Let G=(V,E) be a simple connected graph where $V=\{v_{1},\cdots ,v_{n}\}$ is the set of nodes and E⊂V×V is the set of edges. n=|V| and m=|E| are the number of nodes and edges, respectively. There are two kinds of information from G, i.e.,

- Attribute information: It represents the attribute features carried by the datasets. For example, the signal intensity of a signal tower (which can be abstracted as a node in the network), is a kind of attribute information. Actually, not only nodes but also edges in graphs can contain attribute information. For example, the bonds between molecule pairs can have different types, which can be included in the edge features. We denote the node features by $X={\left(x_{1},\cdots ,x_{n}\right)}^{T}\in R^{n\times d}$ and edge features by $E={\left(x_{e_{1}},\cdots ,x_{e_{m}}\right)}^{T}\in R^{m\times q}$, where *d* and *q* are the dimension of node and edge features, respectively.
- Structure information: It represents the positions and interactions of nodes. Because of the absence of canonical node ordering, without loss of generality, position information can be viewed as a simple kind of interactions between nodes, i.e., a node is adjacent or nonadjacent to others. More complex interactions are simply represented by the node–edge form. Thus, in graphs, structure information is usually encoded by the adjacency matrix of the entire graph or subgraphs. Let $A=\left\{a_{ij}\right\}\in R^{n\times n}$ denote the adjacency matrix, where $a_{ij}=1$ when $(v_{i},v_{j})\in E$, and $a_{ij}=0$ otherwise.

### 3.2. Discrete Ricci Curvature

  The Ricci curvature is originally a geometric notion, which plays a very important role in Riemannian manifold analysis. It quantifies the degree of space bending. For its discrete counterpart, the discretized Ricci curvature measures the connectedness of the neighborhood of two nodes.For the discretization of the Ricci curvature, there are two mainstream forms, i.e., the Ollivier Ricci curvature [25,31] and the Forman Ricci curvature [32]. Since the Ollivier Ricci curvature has more theoretical foundations and depicts inherent structures more intrinsically [33], we applied a limit-free Ollivier Ricci curvature [24,34] as the definition of the DRC.

The Ollivier Ricci curvature is defined on the base of the transportation distance. Firstly, we define the probability distribution of nodes on the graph, which indicates the connections or information flow between one node and others, especially its adjacent neighbors.

**Definition 1.**  ***Probability distribution:*** *For ∀α∈[0,1] and ∀x∈V, the information flow from node x to other nodes y∈V can be defined as a probability distribution on V by*

(1) $$m_{x}^{\alpha }\left(y\right):=\left\{\begin{array}{cc} \alpha , & y=x,\\ \left(1-\alpha \right)\frac{\gamma (w_{xy})}{\sum_{z∼x}\gamma \left(w_{xz}\right)}, & y∼x,\\ 0, & otherwise. \end{array}\right.$$

*where $w_{xy}$ denotes the edge weight on edge (x,y)∈E, y∼x means y is connected with x by an edge, and γ(·) is an arbitrary non-negative real-valued one-to-one function. In our experiments, we set γ(w)=w.*

By the virtue of this definition, $m_{x}^{\alpha }$ extracts the local topology of node *x* on the basis of the graph. The relationship between any two nodes *x* and *y* is proportional to the distance between their neighborhoods, which is defined as the transportation distance between two distributions $m_{x}^{\alpha }$ and $m_{y}^{\alpha }$.

**Definition 2.**  ***Transportation distance:*** *Let A(x,y):V×V→[0,1] be a coupling satisfying*

(2) $$\sum_{y\in V}A\left(x,y\right)=m_{x}^{\alpha }\;\mathrm{and}\;\sum_{x\in V}A\left(x,y\right)=m_{y}^{\alpha }.$$

*Then, the transportation distance between two probability distributions $m_{x}^{\alpha }$ and $m_{y}^{\alpha }$ is defined as*

(3) $$W\left(m_{x}^{\alpha },m_{y}^{\alpha }\right):=\underset{A}{\inf}\sum_{x,y\in V}A\left(x,y\right)d\left(x,y\right),$$

*where d(·,·) is a distance function.*

Here, we leveraged Dijkstra’s shortest path distance as d(·,·) in this work. In order to differentiate topology structures on the basis of graph geometry, the DRC is defined as follows:

**Definition 3.**  **
α *-Ricci curvature:***

(4) $$\kappa_{\alpha }\left(x,y\right)=1-\frac{W(m_{x}^{\alpha },m_{y}^{\alpha })}{d(x,y)},\;\forall \alpha \in \left[0,1\right].$$

***Ollivier Ricci curvature [25]:***

(5) $$\kappa \left(x,y\right)=\lim_{\alpha \to 1}\frac{\kappa_{\alpha }\left(x,y\right)}{1-\alpha }.$$

Note that, in the computation of Ollivier’s Ricci curvature, when the node pair *x* and *y* connect densely, κ(x,y) is larger than the sparsely connected pairs. When computing Ollivier’s Ricci curvature, in order to avoid the limit operation, former works set α to 0.5 [3,22] and utilized $\kappa_{\alpha }$ as an approximation of κ. In this work, we leveraged another limit-free version of Ollivier’s Ricci curvature for computation convenience [34].

**Definition 4.**  *Let B:V×V→R be a coupling function. We simply denote $\mu_{x}^{0}$ as $\mu_{x}$. For any x,y∈V, if B satisfies*

- *B(x,y)>0, while B(u,v)≤0 for u≠x or v≠y;*
- *$\sum_{u,v\in V}B\left(u,v\right)=0$;*
- *$\sum_{v\in V}B\left(u,v\right)=-\mu_{x}\left(u\right)$ for all u≠x;*
- *$\sum_{u\in V}B\left(u,v\right)=-\mu_{y}\left(v\right)$ for all v≠y,*

*then we call B as a∗-coupling between $\mu_{x}$ and $\mu_{y}$.*

**Theorem 1.**  *The ∗-coupling-based Ricci curvature is formulated as:*

(6) $$\kappa^{*}\left(x,y\right)=\frac{1}{d(x,y)}\underset{B}{\sup}\sum_{u,v\in V}B\left(u,v\right)d\left(u,v\right).$$

*and for any x,y∈V, x≠y, the following equation holds:*

(7) $$\kappa^{*}\left(x,y\right)=\kappa \left(x,y\right).$$

(Refer to [34] for proof.)

Thus, $\kappa^{*}$ illustrates the topological characteristic of a graph as an Ollivier Ricci curvature and omits the limit calculation. In our implementation, we leveraged this $\kappa^{*}$ curvature when computing the DRC and denoted the DRC by κ for simplicity. The proof of Theorem 1 can be found in [34]. Algorithm 1 formulates the computation of the DRC.    
**Algorithm 1:** Computation of Discrete Ricci Curvature (DRC)
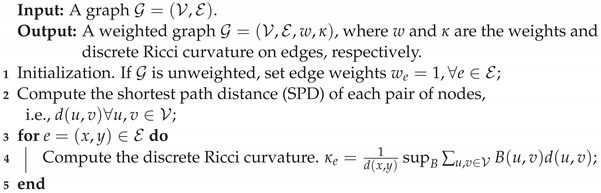


### 3.3. Curvphormer

**Figure 2:**
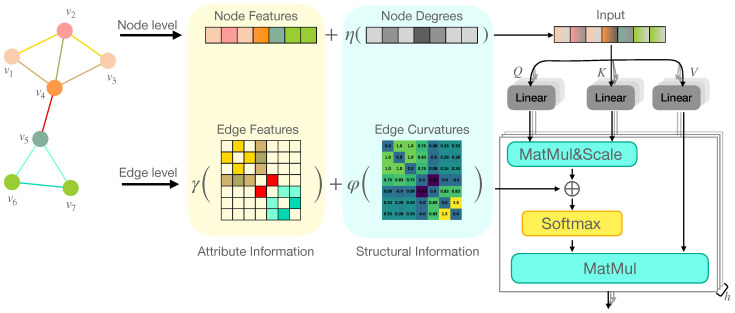
Illustration of Curvphormer with attribute/structure encodings. The input is a combination of two types of node-level information, i.e., node features and node degree encoding. Edge-level information, i.e., encodings of edge features and curvatures, describes the interactions between node pairs; therefore, these two encodings are added to the multihead self-attention module as a bias of the attention weights.

Curvphormer incorporates the advanced geometric information represented by the DRC into a graph-based transformer architecture. The overall architecture of Curvphormer is demonstrated in Figure 2.

#### 3.3.1. Attribute Encoding

As mentioned before, in graph data, the attribute information is the features carried by nodes and edges, describing some specific information in the dataset. Node features are the most important information characterizing a dataset. In Curvphormer, we leveraged the node features without any affine transformation. In many graphs, edges also have attribute features, which are essential for understanding the underlying graph structure. Although edge features are provided by the dataset, they usually indicate the type or intensity of the interactions between nodes. Thus, for any node pair $\left(v_{i},v_{j}\right)$ in a graph, the correlation between $v_{i}$ and $v_{j}$ has to account for the edges connecting them. Let $v_{i}$ and $v_{j}$ be connected by a shortest path denoted by $v_{i}\overset{e_{1}}{∼}\cdots \overset{e_{N}}{∼}v_{j}$. The correlation between $v_{i}$ and $v_{j}$ can be formulated by the mean of the embedded edge features along the path.

(8) $$\gamma \left(v_{i},v_{j}\right)=\frac{1}{N}\sum_{k=1}^{N}{\mathrm{EdgeEmbedding}}_{k}\left(x_{e_{k}}\right),$$

where $x_{e_{k}}\in R^{q}$ is the edge feature of $e_{k}$. ${\mathrm{EdgeEmbedding}}_{k}\left(x_{e_{k}}\right)=x_{e_{k}}^{T}\cdot w_{k}$, $w_{k}\in R^{q}$ is a learnable vector.

#### 3.3.2. Structural Encoding

Structure information here refers to the knowledge of the graph that is induced by the connectedness. As demonstrated in Figure 2, we considered two dimensions of structure information. One is the node-level information to quantify the importance of nodes in the graph. Taking the citation network as an example, the more influential a paper is, the more citations it has, and vice versa. Thus, in an abstract graph, an important node must connect to more neighbors. The node degree is an intuitive choice to describe this node property as in [21]. Let $d_{i}=\sum_{j\in V}a_{ij}$ be the degree of node $v_{i}$. Then, we embed $d_{i}$ into a vector:

(9) $$\eta \left(v_{i}\right)=d_{i}\cdot w_{i},$$

where $w_{i}\in R^{d}$ is a learnable vector. Then, we incorporate the node’s degree embedding matrix $D={\left(\eta \left(v_{1}\right),\cdots ,\eta \left(v_{n}\right)\right)}^{T}\in R^{n\times d}$ with the node features as the input of the subsequent module, i.e., $H^{\left(0\right)}=X+D$.

The other is the edge-level information, which can be interpreted by the positional relationship between any node pairs via the edges connecting them. Former works encoded the position information on graphs by a simple shortest path distance (SPD) [21,35]. However, the SPD can only provide a relative distance on graphs. Graphs can be viewed as a discretized manifold in Riemannian spaces. Thus, the topology structure of the manifold determines the foundation of graphs. A pure SPD neglects the topology structure of the spaces where graphs are embedded in. As we stated in Section 3.2, the DRC depicts the connectedness on the basis of the node’s neighborhoods. Nodes with a positive DRC connect densely, while a negative DRC is related to sparsely connected nodes. By virtue of the expressive power of DRC, we encode the relations of the nodes on the graph topology with

(10) $$\varphi \left(v_{i},v_{j}\right)=\kappa \left(v_{i},v_{j}\right)\cdot w_{ij},$$

where $w_{ij}$ is a learnable scalar.

#### 3.3.3. Self-Attention Mechanism

The self-attention module is the main part of the transformer architecture, which captures the global information by connecting all positions [14,21]. It computes the weighted sum of values, where the weights of values is obtained by a query-key function. Let $H={\left(h_{1},\cdots ,h_{n}\right)}^{T}\in R^{n\times d}$ be the input of the module. In Curvphormer, when a node attends other nodes in the graph, the edge attribute information $\Gamma =\{\gamma \left(v_{i},v_{j}\right)\}$ as well as the DRC-based structural information $\Phi =\{\varphi \left(v_{i},v_{j}\right)\}$ are added to the attention weights to provide more topology-aware ability. Therefore, the self-attention can be formulated as

(11) $$\mathrm{Attention}\left(H\right)=\mathrm{softmax}\left(\frac{QK^{T}}{\sqrt{d_{K}}}+\Gamma +\Phi \right)V,$$

where $Q=HW_{Q},K=HW_{K},V=HW_{V}$, and $W_{Q},W_{K}\in R^{d\times d_{K}},W_{V}\in R^{d\times d_{V}}$. Thus, the correlation between nodes $v_{i}$ and $v_{j}$ is

(12) $$A_{ij}=\mathrm{softmax}\left(\frac{\left(h_{i}W_{Q}\right){\left(h_{j}W_{K}\right)}^{T}}{\sqrt{d_{K}}}+\gamma \left(v_{i},v_{j}\right)+\varphi \left(v_{i},v_{j}\right)\right)V.$$

The multihead self-attention is obtained by

(13) $$\mathrm{MHA}\left(H\right)=\mathrm{Concat}\left({\mathrm{Attention}}_{1}\left(H\right),\ldots ,{\mathrm{Attention}}_{h}\left(H\right)\right)W_{O},$$

where $W_{O}\in R^{hd\times d_{\mathrm{model}}}$.

#### 3.3.4. Curvphormer Structure

Curvphormer follows the basic architecture of Graphormer [21], which is a variant of the vanilla transformer encoder [14]. Each layer of Curvphormer consists of a multihead attention module (MHA) and a feed-forward network (FFN) module. The detailed implementation of a Curvphormer layer is formulated as

(14) $${\hat{H}}^{\left(l+1\right)}=\mathrm{MHA}\left(\mathrm{LayerNorm}\left(H^{\left(l\right)}\right)\right)+H^{\left(l\right)}$$

(15) $$H^{\left(l+1\right)}=\mathrm{FFN}\left(\mathrm{LayerNorm}\left({\hat{H}}^{\left(l+1\right)}\right)\right)+{\hat{H}}^{\left(l+1\right)}$$

Moreover, in order to enhance the ability of Curvphormer to capture the representation of the entire graph, as in [21], a virtual node is applied, which is connected to all nodes in the graph by virtual edges, and the corresponding structural encodings are set to distinct learnable variables.

The training procedure of Curvphormer is mainly based on a transformer encoding module. The self-attention mechanism has a complexity of $O(n^{2}\cdot d)$ per layer, where *n* is the number of nodes, and *d* is the dimension of node features. Before training, Curvphormer computes the DRC as the input of the structural encoding. The computing complexity of DRC is $O(m\cdot {\bar{d}}^{3})$, where *m* is the number of edges, and $\bar{d}$ is the average degree of nodes. It is time-consuming to compute the DRC on very large graphs, thus we compute this valuable structure information of graphs before training.

## 4. Experiments

In this section, we conduct three experiments to intuitively clarify the motivation as well as effectiveness of Curvphormer. Firstly, we illustrate the importance of the topology information in Section 4.1 on a small dataset, i.e., Zachary’s Karate Club Network [36], indicating the importance of our inclusion of the curvature as a factor. Then, we intuitively show the expressiveness of the DRC on graph structures comparing it with the widely used graph structure descriptor SPD in Section 4.2. Finally, we perform experiments on three different scaled real-world datasets to test the performance of Curvphormer in Section 4.3.

### 4.1. Structure Information Is Crucial in Graph-Based Tasks

To illustrate the importance of graph structure information, we devised a binary node classification experiment on the small Karate Club Network (Karate). Karate is composed of two communities with 34 nodes (members of the club). The edges between nodes indicate the interactions between club members. We applied a simple two-layer GCN model [6] to learn the underlying graph structure. Moreover, the node feature was designed based on three cases, i.e., random numbers, the SPD, and the DRC, for testing the influence of different kinds of information in a simple NN-based model.

The accuracy of these three scenarios is shown in Table 1 (best performance in 10 runs). For random features, even though they could not provide any useful information, the classification accuracy was still better than random guess because of the utilization of the adjacency matrix in the model. Notice that when more structure information was provided, the performance of the model improved remarkably. Moreover, the DRC outperformed the SPD in this experiment setting. It indicated that advanced topology information could extract more effective structure information than simple distance information.

### 4.2. Why Does DRC Depict Structure Information Better than SPD?

**Figure 3:**
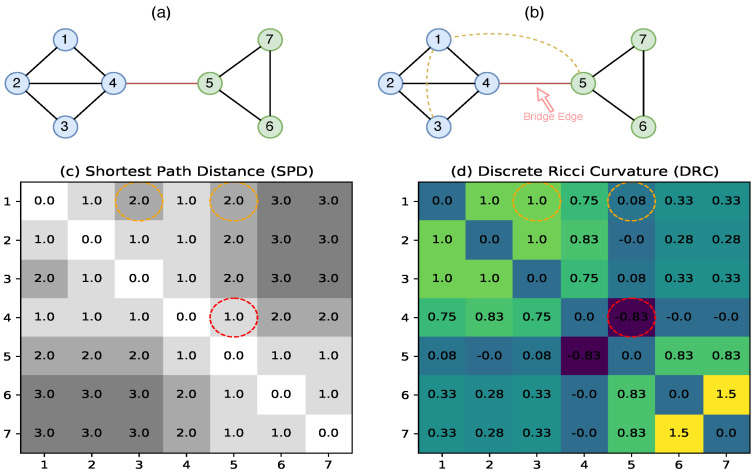
A small graph demonstrates the structural expressiveness of the SPD vs. the DRC. (**a**,**b**) are the graph and hilights. (**c**,**d**) are the SPD and DRC values of node pairs. The difference between (1) inter-/intra-community relations, i.e., 1 and 3 and 1 and 5, (2) the bridge edge $e_{45}$ and other 1-hop pairs, cannot be captured by the SPD but are well described by the DRC.

Now, we intuitively show the expressiveness of the DRC compared to that of the SPD by a small graph composed of two small communities bridged by an edge, as shown in Figure 3. Though both the SPD and DRC had the ability to know there were two communities, the DRC depicted more in-depth structure information than the SPD. Note the interactions between nodes 1,3 and nodes 1,5. Nodes 1 and 3 were from the same community, while nodes 1 and 5 were from different communities. The relationships of these two pairs were different, while ${\mathrm{SPD}}_{13}={\mathrm{SPD}}_{15}=2$ (highlighted by orange circles in Figure 3c). Moreover, edge $e_{45}$ was the only bridge edge connecting the two communities. However, ${\mathrm{SPD}}_{45}=1$ (red dotted circle in Figure 3c) could not differentiate $e_{45}$ from other one-hop pairs. The SPD was incapable of describing these differences in structure. Fortunately, the DRC could amend these defects because it considered the nodes’ neighborhoods. The tightly interacting pairs tended to have a larger DRC than sparsely interacting pairs. ${\mathrm{DRC}}_{13}=1$ was apparently larger than ${\mathrm{DRC}}_{15}=0.08$ for the first case. Meanwhile, ${\mathrm{DRC}}_{45}=-0.83$ highlighted the difference of this edge from others.

### 4.3. Experiments on Real-Word Datasets

**Figure 4:**
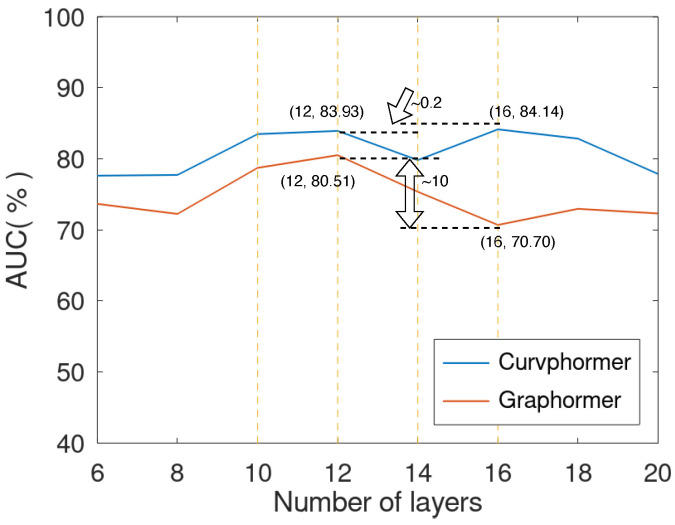
Testing the performance of Curvphormer on MolHIV for different number of layers. Curvphormer surpasses the baseline Graphormer by a significant margin and attains stable satisfactory performance for a varying number of layers.

In this part, we devised our experiments on three different scaled datasets, i.e., MolHIV (small), ZINC (medium), and PCQM4M-LSC (large). Statistics of the datasets are summarized in Table 2. We summarize the statistics of datasets used in this work in Table 1 and Table 3, and Figure 4.

#### 4.3.1. Experimental Set-Up

We benchmarked Curvphormer with the non-topology-aware Graphormer baseline [21]. The basic setting of Curvphormer followed [21] but we modified some parameters for the model fine-tuning. The number of attention heads and the dimension of node/edge features were set to 16. We used AdamW as the optimizer and set the hyperparameter Adam-ϵ to 1 × 10${}^{-8}$ and Adam-$\left(\beta_{1},\beta_{2}\right)$ to (0.99,0.999). The learning rate was set to 2 × 10${}^{-4}$ with a lower bound of 1 × 10${}^{-9}$. The batch size was set to 512. All models and tasks were trained on eight NVIDIA 3080ti GPUs for about three days. Other settings were the same as those of the baseline. We trained Curvphormer on PCQM4M-LSC and ZINC from scratch. We fine-tuned the pretrained model on ZINC with the small dataset MolHIV to test the transferable ability of Curvphormer. In addition, in order to test if Curvphormer could effectively resist the performance drop caused by oversmoothing, we tested Curvphormer on the MolHIV dataset with a varying number of layers up to 20.

#### 4.3.2. Results

Table 3 summarizes the performance of Curphormer and other baselines on PCQM4M-LSC, ZINC, and MolHIV. The metrics were the mean absolute error (MAE) for the regression task and the AUC for the classification task. We report the MAE on the validation set (ValidMAE) for PCQM4M-LSC because its test set was not publicly available. Curvphormer achieved the best results and noticeably surpassed the previous state-of-the-art GNNs as well as the recent graph-transformer model GT [18] and Graphormer [21].

Next, we tested Curvphormer’s performance further on the MolHIV dataset by comparing it with the baseline Graphormer. Figure 4 shows that both models were capable of resisting oversmoothing. Meanwhile, Curvphormer surpassed Graphormer by a noticeable margin for all layer configurations. It is noteworthy that when the model layer changed from 12 to 16, the performance of Graphormer dropped from 80.51 to 70.70. In contrast, Curvphormer achieved a comparable result after a slight drop.

## 5. Conclusions and Discussion

This work introduced Curvphormer, a topology-aware graph transformer that incorporates advanced structure information into an expressive Graphormer architecture. The DRC effectively differentiated the topology structure of graphs with the homophily property and helped our model achieve remarkable performance improvements on different scaled datasets in graph classification/regression tasks. It showed that applying more geometric descriptors to expressive graph models is rewarding. Meanwhile, the exploration of graph structure information is still challenging. For example, discovering the topology information of heterogeneous graphs still needs future endeavors. Moreover, the computation complexity of the DRC restricts its application in large dynamic systems. In a nutshell, Curvphormer inspires a better understanding of graph structure and encourages future work.

## Figures and Tables

**Table 1:** Test on different types of structure information on the Karate dataset with a 2-layer GCN. Structure information yields better results, and advanced topological DRC outperforms SPD.

Feature Type	Feature Description	Accuracy (%)
Random numbers	No useful information	78
SPD	Provides distance information for nodes	95
DRC	Provides advanced topology information	**97**

**Table 2:** Statistics of the datasets.

DATASETS	Scale	#Graphs	#Nodes	#Edges	Task Type
ZINC (sub-set)	Small	12,000	277,920	597,960	Regression
MolHIV	Medium	41,127	1,048,738	1,130,993	Binary classification
PCQM4M-LSC	Large	3,803,453	53,814,542	55,399,880	Regression

**Table 3:** Results on the PCQM4M-LSC, ZINC, and MolHIV datasets. The performance metric for the regression task on PCQM4M-LSC and ZINC is the MAE, and the AUC for the classification task on MolHIV. validMAE and testMAE refer to the MAE on the validation set and test set, respectively. The test set of PCQM4M-LSC is not publicly available. Curvphormer outperforms the benchmarks on all these datasets.

Datasets	Scale	Task	Model	#Layers	#Param	validMAE
PCQM4M-LSC	Large	Regression	GCN [37]	12	2.0M	0.1691
			GIN [38]	12	3.8M	0.1537
			DeeperGCN [39]	12	25.5M	0.1398
			GT [18]	12	0.6M	0.1400
			GraphormerSMALL [21]	12	12.5M	0.1264
			Graphormer [21]	12	47.1M	0.1234
			**Curvphormer**	8	34.1M	**0.1024**
			Model	#Layers	#Param	testMAE
ZINC	Medium	Regression	GIN [38]	2	510K	0.526
			GraphSage [8]	2	505K	0.398
			GAT [7]	2	531K	0.384
			GCN [37]	2	505K	0.367
			GatedGCN-PE [40]	2	505K	0.367
			PNA [41]	16	387K	0.214
			GraphormerSLIM [21]	12	489K	0.122
			**Curvphormer**	8	34.1M	**0.080**
			Model	#Layers	#Param	AUC (%)
MolHIV	Small	Classification	GCN-GraphNorm [37]	12	526K	78.83
			PNA [41]	12	326K	79.05
			PHC-GNN [42]	12	111K	79.34
			DeeperGCN–FLAG [39]	12	532K	79.42
			DGN [43]	12	114K	79.70
			Graphormer-FLAG [21]	12	47.0M	80.51
			**Curvphormer**	12	47.1M	**83.93**

## Data Availability

Data sharing not applicable.
